# A comparison of two pencil beam scanning treatment planning systems for proton therapy

**DOI:** 10.1002/acm2.12235

**Published:** 2017-12-04

**Authors:** Ulrich W. Langner, Michelle Mundis, Dan Strauss, Mingyao Zhu, Sina Mossahebi

**Affiliations:** ^1^ Maryland Proton Treatment Center University of Maryland Baltimore MD USA

**Keywords:** Monte Carlo methods, treatment planning

## Abstract

**Objective:**

Analytical dose calculation algorithms for Eclipse and Raystation treatment planning systems (TPS), as well as a Raystation Monte Carlo model are compared to corresponding measured point doses.

**Method:**

The TPS were modeled with the same beam data acquired during commissioning. Thirty‐five typical plans were made with each planning system, 31 without range shifter and four with a 5 cm range shifter. Point doses in these planes were compared to measured doses.

**Results:**

The mean percentage difference for all plans between Raystation and Eclipse were 1.51 ± 1.99%. The mean percentage difference for all plans between TPS models and measured values are −2.06 ± 1.48% for Raystation pencil beam (PB), −0.59 ± 1.71% for Eclipse and −1.69 ± 1.11% for Raystation monte carlo (MC). The distribution for the patient plans were similar for Eclipse and Raystation MC with a *P*‐value of 0.59 for a two tailed unpaired *t*‐test and significantly different from the Raystation PB model with *P* = 0.0013 between Raystation MC and PB. All three models faired markedly better if plans with a 5 cm range shifter were ignored. Plan comparisons with a 5 cm range shifter give differences between Raystation and Eclipse of 3.77 ± 1.82%. The mean percentage difference for 5 cm range shifter plans between TPS models and measured values are −3.89 ± 2.79% for Raystation PB, −0.25 ± 3.85% for Eclipse and 1.55 ± 1.95% for Raystation MC.

**Conclusion:**

Both Eclipse and Raystation PB TPS are not always accurate within ±3% for a 5 cm range shifters or for small targets. This was improved with the Raystation MC model. The point dose calculations of Eclipse, Raystation PB, and Raystation MC compare within ±3% to measured doses for the other scenarios tested.

## 1 INTRODUCTION

Many proton centers around the world are implementing pencil beam spot scanning as a preferred delivery method for proton therapy. Spot scanning beams have the ability to modulate energy as well as intensity without the use of apertures/collimators and compensators in the beamline.^1^ At our center spot scanning techniques are exclusively used. When considering how to most effectively treat patients with proton therapy, different treatment planning system provide different advantages and disadvantages.^2^, ^3^ These differences in different treatment planning systems were discussed in various articles for photons^4^, ^5^, ^6^, ^7^, ^8^ and for protons.^9^, ^10^, ^11^ Pencil beam scanning can also be simulated with Monte Carlo models,^12^ although this is more time consuming and not practical for everyday treatment planning. At our center we use two commercially available clinical treatment planning systems (TPS), Eclipse and Raystation. In this work, we compare the analytical dose calculation algorithms for these systems, as well as for a Raystation Monte Carlo model to corresponding measured point doses. The main issues using analytical algorithms for range shifters are that the average proton energy decreases when scattering angle increases during the non‐elastic scattering processes as shown in Lin et al.,^12^ especially for small fields or fields including a range shifter.^10^, ^11^ We therefore compare the planning systems to quantify these inaccuracies.

## 2 MATERIALS AND METHODS

The treatment planning system (TPS) and proton pencil beam calculation models used are Varian Eclipse v11^13^ (Varian Medical Systems, Palo Alto, CA, USA), Raystation v5 pencil beam (PB), and Raystation v6 Monte Carlo (MC) (RaySearch Medical Laboratories AB, Stockholm, Sweden).^14^, ^15^, ^16^, ^17^, ^18^ The TPS were modeled with the same beam data acquired during commissioning.^19^, ^20^ To commission the TPS the integrated depth dose curves (IDDs) and spot profiles in air were measured over the entire range of energies available, starting from 245 MeV, 240 MeV, and then every 10 MeV down to 70 MeV. For both TPS only the spot profiles at one gantry angle could be used for commissioning, even though there is a change in full width half maximum of the spots as function of gantry angle.^20^ We choose to use the spot profiles measured at gantry 0 every 10 MeV from 70 MeV to 245 MeV, and verified that the variation at other angles and among all treatment rooms are within 15%. In addition to the spot profiles in air at isocenter, they were also measured for both TPS at 10 cm and 20 cm above and below isocenter as well as for each range shifter commissioned. Range shifters provide dose coverage for more superficial tumors. For both TPS the 5.7 cm, 3.42 cm and 2.28 cm water equivalent thickness (WET) range shifters were commissioned. The range shifters were commissioned with an airgap of 26 cm. This distance is an approximation of what will be used clinically for most cases. Smaller airgaps will be used during planning, but might not always be possible because of patient couch collisions as the snout is extended.

Absolute calibration was done using the TRS398 protocol.^21^ For Eclipse the absolute dose measurement was done at a water equivalent depth of 2 cm for all the energies measured for the IDDs. Raystation required measurements of absolute dose at a water equivalent depth at the position between 1 cm depth and half way between maximum of the Bragg peak for the same energies. The resulting absolute IDDs from each TPS differed 0.30 ± 0.25% at 2 cm depth.

Eclipse used a depth dose normalization table to correct for discrepancies between measurements and doses calculated by the TPS. Measurements for this table are made in the center of the spread out Bragg peak (SOBP) at predetermined ranges and SOBPs and compared to calculated doses for the same setup. The ratio of the calculations and measurements are then entered as correction factors in a look‐up table. Raystation did not have a similar tool.

To verify the modeled TPS measurements, 35 typical plans were made with the TPS, 31 without a range shifter and four with a 5 cm range shifter (5.7 cm WET). A 40 cm water equivalent virtual phantom was created with the isocenter placed at 20 cm depth. The Hounsfield units for this virtual phantom was forced to 0 for Eclipse, resulting in a mass density of 1.024 g/cm^3^ and a relative stopping power of 1.002. For Raystation the same CT calibration curve was used and the phantom material was set as water, resulting in a mass density of 1.00 g/cm^3^. First generic water equivalent phantom plans were created for targets close to the surface, deeper in the phantom and at a midrange. Small targets (2 × 2 × 2 cm^3^) and large targets (15 × 15 × 15 cm^3^) were created at each position. Other plans are fields of 10 × 10 cm^2^ with variable range and SOBP. The 5 cm range shifter was also used for targets close to the surface to evaluate equivalence between Raystation and Eclipse for plans with range shifters. These plans were first calculated in Eclipse and then exported to Raystation, where the same spot distribution was recalculated with the Raystation dose algorithm for the same weights and spot placements. This was done to test dosimetric equivalence under the same conditions for each TPS. The Raystation MC calculation was done with 1% statistical uncertainty. To evaluate clinical plans, the same was done with 28 arbitrary patient fields, 14 without range shifter, 2 with a 2 cm range shifter, and 12 with a 5 cm range shifter. The absolute point dose values obtained from each TPS for these various plans were then compared with the measurements and each other. Measurements were made in a water tank with the isocenter placed at 20 cm depth, i.e., the source surface distance was 206 cm. A small volume ion chamber (0.04 cc) was used and the measurement depth of the ion chamber was determined for each plan in order to place the chamber center in the center of the spread out Bragg peak for each field.

## 3 RESULTS

**Figure 1:**
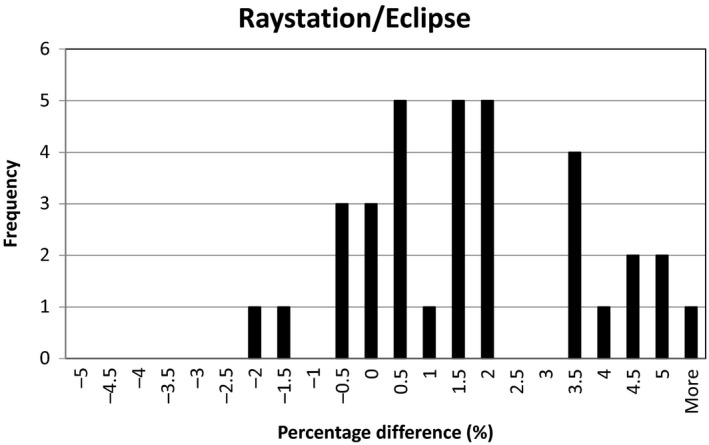
Percentage difference distribution for plans calculated with Raystation TPS and Eclipse TPS.

**Figure 2:**
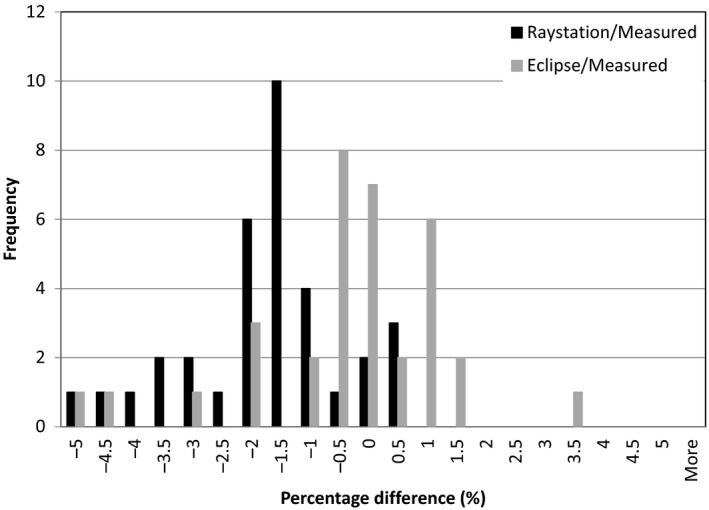
Percentage difference distribution of point doses for plans calculated with the Raystation TPS and measured doses and with Eclipse TPS and measured doses.

Table 1, Figs. 1 and 2 illustrate the percentage differences between point doses calculated with Raystation and corresponding measured point doses and Eclipse as well as the corresponding measured point doses. The mean percentage difference for all plans between Raystation PB and Eclipse were 1.51 ± 1.99%. The mean percentage difference for all plans between TPS models and measured values are −2.06 ± 1.48% for Raystation PB, −0.59 ± 1.71% for Eclipse and −1.69 ± 1.11% for Raystation MC. The mean percentage difference between plans excluding the plans with the 5 cm range shifter and the plans with a range of 35 cm are −1.45 ± 0.86% between Raystation PB and the measured values, −1.07 ± 1.21% between Eclipse and the measured values, and 1.67 ± 1.07% between Raystation MC and the measured values. The percentage difference between Raystation PB and Eclipse for these plans were 0.39 ± 1.12%.

**Table 1:** Measured and calculated doses for plans calculated in Eclipse and recalculated in Raystation and with Raystation monte carlo

Plan (R = range; S = SOBP; SI* ×* LR* ×* AP)	Nominal range (cm)	WED of measurement (cm)	Raystationdose (cGy)	Eclipse dose (cGy)	Raystation MC dose (cGy)	Measured dose (cGy)	% Difference Raystation and Eclipse	% Difference Raystation and measured	% Difference Eclipse and measured	% Difference Raystation MC and measured
Open snout (i.e., no range shifter)										
10 × 10 × 10 cm^3^ volume	25.16	20.00	200.50	197.60	202.00	196.46	1.47	−2.01	−0.58	−2.82
10 × 10 × 10 cm^3^ volume	15.10	9.85	219.10	218.40	219.00	216.90	0.32	−1.00	−0.69	−0.97
3 × 10 × 10 cm^3^ volume	25.16	19.87	197.20	197.75	196.00	193.46	−0.28	−1.90	−2.17	−1.31
3 × 10 × 10 cm^3^ volume	15.10	9.85	217.00	218.40	216.00	216.84	−0.64	−0.07	−0.72	0.39
2 × 2 × 2 cm^3^ volume	9.02	6.59	220.50	224.22	221.00	213.62	−1.66	−3.12	−4.73	−3.46
R10S1	10.00	2.28	41.70	41.76	43.00	41.76	−0.14	0.13	−0.01	−2.98
R10S5	10.00	7.68	110.50	111.25	109.00	108.59	−0.68	−1.73	−2.39	−0.38
R10S10	10.00	7.08	110.60	111.70	110.00	108.94	−0.98	−1.51	−2.48	−0.98
R15S1	15.00	6.78	40.00	40.06	40.00	40.01	−0.15	0.01	−0.14	0.01
R15S5	15.00	12.58	107.20	106.15	107.00	105.43	0.99	−1.65	−0.68	−1.49
R15S10	15.00	9.58	109.20	109.10	110.00	108.08	0.09	−1.03	−0.94	−1.78
R15S15	15.00	9.08	110.00	109.80	110.00	108.64	0.18	−1.24	−1.06	−1.25
R20S1	20.00	12.58	40.00	40.84	40.00	39.41	−2.05	−1.47	−3.49	−1.49
R20S5	20.00	17.88	102.70	101.60	103.00	100.80	1.08	−1.85	−0.79	−2.18
R20S10	20.00	15.00	330.20	330.00	330.20	330.36	0.06	0.05	0.11	0.05
R20S15	20.00	15.08	108.90	106.82	107.00	106.26	1.95	−2.42	−0.52	−0.69
R20S20	20.00	15.08	108.00	107.70	109.00	107.14	0.28	−0.80	−0.52	−1.74
R25S1	25.00	13.08	36.70	36.28	37.00	35.75	1.16	−2.58	−1.45	−3.48
R25S5	25.00	22.48	97.70	96.40	99.00	95.98	1.35	−1.76	−0.43	−3.14
R25S10	25.00	20.88	100.30	98.70	101.00	98.64	1.62	−1.66	−0.06	−2.39
R25S15	25.00	20.88	103.00	101.30	102.00	101.01	1.68	−1.93	−0.28	−0.98
R25S20	25.00	20.88	107.00	105.25	107.00	104.95	1.66	−1.91	−0.29	−1.95
R25S25	25.00	20.88	107.20	105.45	107.00	105.20	1.66	−1.86	−0.24	−1.71
R35S1^a^	35.00	20.08	34.50	32.90	34.00	33.07	4.86	−4.15	0.51	−2.82
R35S5^a^	35.00	20.08	51.20	48.92	50.00	49.32	4.66	−3.67	0.82	−1.38
R35S10^a^	35.00	20.08	82.50	79.02	81.00	79.32	4.40	−3.85	0.38	−2.11
R35S15^a^	35.00	26.08	101.70	98.31	101.00	99.20	3.45	−2.46	0.91	−1.81
R35S20^a^	35.00	26.08	103.00	99.69	103.00	100.70	3.32	−2.23	1.01	−2.28
R35S25^a^	35.00	26.08	105.00	101.59	105.00	102.58	3.36	−2.31	0.97	−2.36
R35S30^a^	35.00	26.08	110.90	107.29	110.00	108.26	3.36	−2.38	0.91	−1.60
RS 5 cm^b^										
10 × 10 × 2 cm^3^ volume^b^	7.01	6.46	227.40	218.19	220.00	219.50	4.22	−3.48	0.60	−0.23
2 × 2 × 2 cm^3^ volume^b^	9.02	6.46	220.80	217.60	214.00	204.98	1.47	−7.16	−5.80	−4.40
10 × 20 × 5 cm^3^ volume^b^	4.76	2.56	241.10	227.75	233.00	230.20	5.86	−4.52	1.07	−1.22
15 × 15 × 15 cm^3^ volume^b^	27.75	20.00	202.10	195.20	202.00	201.28	3.53	−0.41	3.11	−0.36
All plans						Mean	1.51	−2.06	−0.59	−1.69
						Std	1.99	1.48	1.71	1.11
All plans (without^a^,^b^)						Mean	0.39	−1.45	−1.07	1.67
						Std	1.12	0.86	1.21	1.07
Only^b^						Mean	3.77	−3.89	−0.25	1.55
						Std	1.82	2.79	3.85	1.95

If only the plans with a 5 cm range shifter are evaluated, the percentage difference between Raystation PB and Eclipse is 3.77 ± 1.82%, while the absolute percentage difference between the TPS and the measured values increases to −3.89 ± 2.79% for Raystation PB, −0.25 ± 3.85% for Eclipse, and 1.55 ± 1.95% for Raystation MC. Even though the mean percentage difference between Eclipse and the measured values for the plans with the range shifter is small, two of the four plans had percentage differences in more than 3% and the measured doses are mostly higher than the TPS doses. For Raystation PB plans the measured doses are lower than the TPS doses for all the plans and 3 of the 4 plans have percentage differences higher than 3%, with a maximum of 7.16%. Comparing the TPS doses, the Raystation PB plans with a 5 cm range shifter give doses higher than those calculated by Eclipse by more than 3% for 3 of the 4 plans. This can in part be due to the slightly lower mass density of 1.00 g/cm^3^ assigned for the water phantom in Raystation, compared to 1.024 g/cm^3^ in Eclipse (for HU of 0), resulting in a difference in stopping power in water compared to Eclipse.

**Figure 3:**
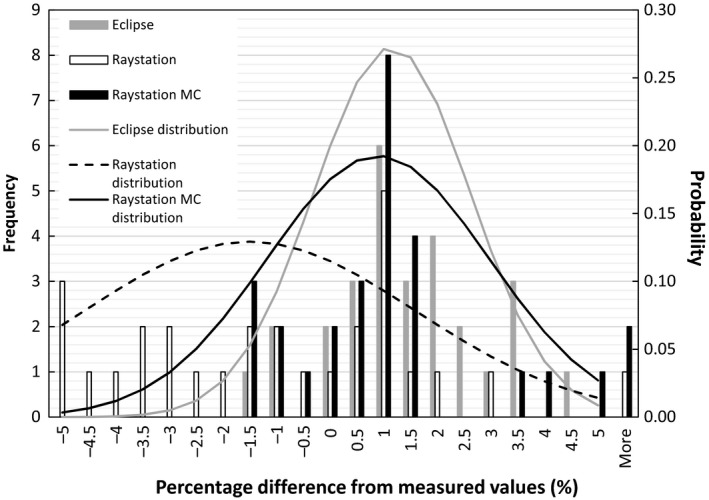
Percentage difference distribution of point doses for plans calculated with Eclipse, Raystation, and Raystation Monte Carlo TPS models compared to measured doses for the same plans. The lines represents normal distributions based on the mean and standard deviation of each distribution.

In Fig. 3 and Table 2 a percentage difference distribution is shown of point doses for 28 patient QA plans calculated with Eclipse, Raystation PB, and Raystation MC TPS models compared to measured doses for the same plans. The sites used for each of these plans are given in Table 2. Normal distributions based on the mean and standard deviation of each distribution are also shown in Fig. 3. From these data follows that the distributions of doses calculated with Eclipse and Raystation MC compared to measurements were similar with a mean and standard deviation of 1.15 ± 1.46% for Eclipse and 0.90 ± 2.07% for Raystation MC. The Raystation PB doses compared to measurements had a mean and standard deviation of −1.50 ± 3.08%.

**Table 2:** Measured and calculated doses for patient plans for various sites calculated in Eclipse and recalculated in Raystation and with Raystation monte carlo

Site	Range shifter	Eclipse dose (cGy)	Raystation dose (cGy)	Raystation mc dose (cGy)	Measured dose (cGy)	% Difference eclipse (%)	% Difference Raystation (%)	% Difference Raystation MC (%)
Esophagus^a^	No	61.18	63.64	62.73	63.14	3.10	−0.79	0.65
Esophagus^a^	No	80.82	81.82	81.82	82.21	1.69	0.48	0.48
Esophagus^a^	No	79.09	78.18	78.18	78.70	−0.50	0.66	0.66
Abdomen^a^	No	56.09	56.36	56.36	56.74	1.14	0.66	0.66
Abdomen^a^	No	53.09	53.64	54.55	54.57	2.71	1.71	0.04
Abdomen^a^	No	53.55	54.55	54.55	53.94	0.73	−1.12	−1.12
Rectum^b^	5 cm	65.18	69.09	67.27	67.54	3.49	−2.30	0.40
Rectum^b^	5 cm	38.27	40.00	39.09	38.34	0.18	−4.33	−1.96
Rectum^b^	5 cm	42.91	46.36	44.55	44.86	4.35	−3.35	0.70
Rectum^b^	5 cm	36.64	38.18	37.27	37.13	1.33	−2.83	−0.38
Rectum^b^	5 cm	35.27	36.36	35.45	35.83	1.56	−1.49	1.05
Rectum^b^	5 cm	36.36	38.18	37.27	36.93	1.53	−3.39	−0.93
Groin^b^	5 cm	89.55	95.45	86.78	91.07	1.67	−4.81	4.71
Groin^b^	5 cm	88.82	96.36	87.60	90.87	2.26	−6.05	3.59
Hip^a^	No	49.27	48.18	48.18	48.54	−1.51	0.74	0.74
Hip^a^	No	51.18	50.91	50.91	51.62	0.85	1.38	1.38
Liver^b^	5 cm	163.82	170.91	155.37	165.11	0.78	−3.51	5.90
Liver^b^	5 cm	156.55	163.64	148.76	157.85	0.83	−3.67	5.76
Pancreas^a^	No	48.45	48.18	49.09	49.60	2.31	2.86	1.03
Pancreas^a^	No	47.82	48.18	48.18	48.51	1.43	0.68	0.68
Pancreas^a^	No	47.27	46.36	47.27	48.90	3.33	5.19	3.33
Pancreas^a^	No	59.27	59.09	59.09	59.44	0.28	0.59	0.59
Pelvis^a^	No	79.82	79.09	80.00	78.87	−1.20	−0.28	−1.43
Pelvis^a^	No	78.09	78.18	77.27	78.27	0.23	0.11	1.27
Rectum^a^	2 cm	57.73	59.09	59.09	58.13	0.69	−1.65	−1.65
Rectum^a^	2 cm	57.73	59.09	58.18	58.13	0.69	−1.65	−0.09
Axilla^b^	5 cm	182.36	191.82	183.00	179.69	−1.49	−6.75	−1.84
Axilla^b^	5 cm	187.91	204.55	185.95	187.56	−0.19	−9.06	0.86
All plans					Average	1.15	−1.50	0.90
					Std	1.46	3.08	2.07
					Min	−1.51	−9.06	−1.96
					Max	4.35	5.19	5.90
Only^a^ (no 5 cm RS)					Average	1.00	0.60	0.45
					Std	1.41	1.71	1.19
					Min	−1.51	−1.65	−1.65
					Max	3.33	5.19	3.33
Only^b^					Average	1.36	−4.29	1.49
					Std	1.57	2.11	2.82
					Min	−1.49	−9.06	−1.96
					Max	4.35	−1.49	5.90

For the 16 patient plans not containing the 5 cm range shifter, the mean and standard deviations decreased markedly, i.e., 1.00 ± 1.41% for Eclipse, 0.60 ± 1.71% for Raystation PB, and 0.45 ± 1.19% for Raystation MC. This was true especially for both the Raystation models. For the 12 patient plans including a 5 cm range shifter, the mean and standard deviation were 1.36 ± 1.57% for Eclipse, −4.29 ± 2.11% for Raystation v5, and 1.49 ± 2.82% for Raystation MC.

**Figure 4:**
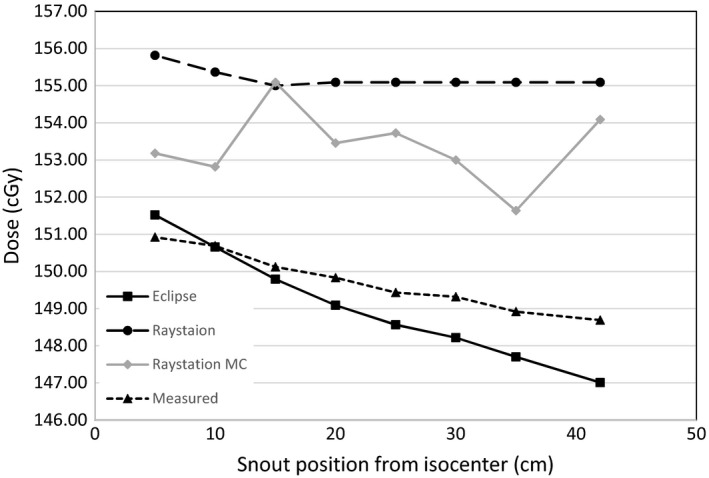
Point doses for 10 × 10 × 10 cm^3^ volumes in water with a range of 20 cm and a SOBP of 10 cm calculated at the center of the SOBP with Eclipse, Raystation, and Raystation Monte Carlo TPS models compared to measured doses for the same plans for different position of the range shifter from isocenter. The plan was initially calculated with a 5 cm range shifter at 42 cm from isocenter. The plan dose was then recalculated without changing the spot distribution for different range shifter positions from isocenter.

In Fig. 4 point doses for 10 × 10 × 10 cm^3^ volumes in water with a range of 20 cm and a SOBP of 10 cm calculated at the center of the SOBP with Eclipse, Raystation PB, and Raystation MC TPS models were compared to measured doses for the same plans for different position of the range shifter from isocenter. This was done in order to verify how different airgaps will affect the dose calculations for range shifters, since the spot profiles were measured during commissioning with a 26 cm airgap. Only spot profiles for one airgap position could be used for modeling in the TPS, so 26 cm was chosen to be an approximation of the airgap for most patient treatments. The airgap can mostly not be much smaller to avoid collisions with the patient or couch. The plan was initially calculated with a 5 cm range shifter at 42 cm from isocenter. The plan dose was then recalculated without changing the spot distribution for different range shifter positions from isocenter. The absolute percentage difference between calculated and measured doses increased as the distance from isocenter increased for the Raystation plans with a maximum of 4.3% and 3.6%, respectively, for the Raystation PB and Raystation MC models. The calculated doses for the Raystation PB model also stayed constant from an airgap of 15 cm to the maximum airgap. For the eclipse plans the maximum absolute percentage difference is 1.1% and the calculated doses decreased as the airgap increased, similar to the measured doses, although it decreased faster.

## 4 DISCUSSION

From Table 1 it follows that none of the TPS were accurate with calculations with a 5 cm range shifter, although the Raystation MC model did slightly better in this scenario with a smaller standard deviation. If a 3% tolerance for agreement of point dose is used as metric then most of the Raystation MC plans were within tolerance. The calculated plan doses for a range of 35 cm also differed by more than 3% between Raystation and Eclipse, although both were within 2.5% from the measured values, with Eclipse doses closer to the measured values than those of Raystation.

Neither of the analytical calculation models of the TPSs were accurate in calculating dose for plans with a 5 cm range shifter, with Eclipse calculating doses mostly lower than measured doses and Raystation calculating doses higher than measured doses. This indicates that neither TPS model the additional scattering in the range shifter correctly. The Raystation MC model alleviates this problem with a calculated dose mostly lower than the measured dose, indicating that scatter in the range shifter is still underestimated. This can also be seen in the smaller mean and standard deviations if the range shifter plans are excluded, especially for both the Raystation models.

Both TPS had calculated doses more than 3% higher for a small shallow target of 2 × 2 × 2 cm^3^ dimension. The same is not true for targets that are small but deeper, since plans with ranges larger than 10 cm and a SOBP of 1 cm were within 2%. Raystation PB point doses for plans with a range of 35 cm or larger were also not as accurate as for the other TPS used.

The distribution for the patient plans were similar for Eclipse and Raystation MC with a *P*‐value of 0.59 for a two tailed unpaired *t*‐test and significantly different from the Raystation PB model with *P* = 0.0013 between Raystation MC and PB. Again the models faired markedly better if plans with a 5 cm range shifter were ignored. At least 68% of the plans would then pass comparisons within a 3% tolerance for all the TPS. The Raystation MC model markedly improved the dose calculation for the 5 cm range shifter, although it gave similar results to the analytical methods for fields without a 5 cm range shifter.

As the difference in snout position increased from the value for which it was calculated in the TPS, the spot size will change and an increase or decrease in measured dose is expected. The calculated dose in the Raystation PB and MC plans showed very little variation as function of snout position and had the largest difference from measured doses, increasing as the airgap increased. Eclipse correlated the best with the measured doses, although the increase in dose was faster than for the measured doses as the range shifter came closer to isocenter. It was expected that the coincidence of measured and calculated values would occur at a snout position of 26 cm, since that is where the measurements were taken during commissioning. Neither TPS thus modeled the airgap satisfactorily. A more accurate modelling of the primary proton energy spectrum is needed. Measurements for additional airgaps during commissioning might increase the correspondence of measured and calculated doses if it can be incorporated in the TPS models.

## 5 CONCLUSION

Both Eclipse and Raystation PB TPS are not always accurate within 3% for a 5 cm range shifters or for small targets compared to measurements. This was improved with the Raystation MC model. Dose measurements and accompanying adjustments are thus recommended when range shifters are used or for small or shallow targets. The Raystation Monte Carlo model should be used for these scenarios if possible. The point dose calculations of Eclipse, Raystation PB, and Raystation MC compare within 3% to measured point doses for the other scenarios tested and all the models give acceptable results. Calculations for plan comparison measurements were done in a virtual water phantom and does not include the accuracy of calculation in a heterogeneous medium, although both TPS passed the IROC lung phantom accreditation tests. Although not always possible, an airgap during treatment as close as possible to the air gap used for measuring spot profiles during commissioning should be used to obtain the best accuracy.

## CONFLICT OF INTEREST

The authors declare no conflict of interest.
